# Intelligent Structured Intermittent Auscultation (ISIA): evaluation of a decision-making framework for fetal heart monitoring of low-risk women

**DOI:** 10.1186/1471-2393-14-184

**Published:** 2014-05-31

**Authors:** Robyn M Maude, Joan P Skinner, Maralyn J Foureur

**Affiliations:** 1 Graduate School of Nursing, Midwifery and Health, Victoria University of Wellington, Newtown, PO Box 7625, Wellington, New Zealand; 2 Centre for Midwifery, Child and Family Health, Faculty of Health, University of Technology, Sydney, Australia

**Keywords:** Intermittent auscultation, Fetal heart rate monitoring, Decision-making, Clinical, Knowledge translation, Mixed methods

## Abstract

**Background:**

Research-informed fetal monitoring guidelines recommend intermittent auscultation (IA) for fetal heart monitoring for low-risk women. However, the use of cardiotocography (CTG) continues to dominate many institutional maternity settings.

**Methods:**

A mixed methods intervention study with before and after measurement was undertaken in one secondary level health service to facilitate the implementation of an initiative to encourage the use of IA. The intervention initiative was a decision-making framework called Intelligent Structured Intermittent Auscultation (ISIA) introduced through an education session.

**Results:**

Following the intervention, medical records review revealed an increase in the use of IA during labour represented by a relative change of 12%, with improved documentation of clinical findings from assessments, and a significant reduction in the risk of receiving an admission CTG (RR 0.75, 95% CI, 0.60 – 0.95, p = 0.016).

**Conclusion:**

The ISIA informed decision-making framework transformed the practice of IA and provided a mechanism for knowledge translation that enabled midwives to implement evidence-based fetal heart monitoring for low risk women.

## Background

The monitoring of fetal well-being during labour and birth is a central component of modern day midwifery care. Intermittent auscultation (IA), or listening to and counting the sounds of the fetal heart beat, is a suitable monitoring method for ‘low risk’ women (well women with uncomplicated pregnancies), and a fundamental midwifery skill. This low-tech monitoring modality requires the midwife to remain close by the woman throughout labour and to be in physical contact in order to monitor the well-being of the unborn baby. IA requires effective communication as well as the ability to listen carefully and interpret heart sounds. However, IA is a skill that is rapidly disappearing from midwifery practice because of the preferential application of an electronic and continuous means of listening to the fetal heart using a cardiotocograph (CTG) machine.

The use of CTG monitoring, for assessment and screening on admission to the maternity unit (admission CTG) and continuously during labour, has increased in the United States and Canada from 62% to around 93% in the past two decades [1,2]. The increased use of admission CTG and continuous CTG monitoring for low-risk women in the absence of clinical indications is of particular concern as it has been shown to have detrimental effects [3]. Systematic reviews of IA compared with admission CTG have revealed increased interventions such as epidural analgesia, continuous CTG monitoring, fetal blood sampling, and an increased risk of caesarean section and instrumental delivery in women receiving admission CTG, without any improvements in neonatal outcomes [4,5]. The admission CTG has been shown to have poor predictive value of adverse fetal outcomes, a high rate of error, falsely identifies 'fetal distress' and offers no benefit in low-risk women [4].

Fetal monitoring experts Gibb & Arulkumaran write: “[Excessive] technology should not be applied to those [women] who are manifestly at low-risk. It may confer no benefit, can generate both non-medical and medical anxiety, and through subtle effects may cause significant harm” [6] (p.vii). Further, the CTG monitor becomes the centre of attention for family and caregivers rather than the woman herself [3,7] and presents a physical barrier to the provision of ‘hands-on’ support to the labouring woman. Additionally, the woman’s confidence in her ability to give birth safely without the use of technology is diminished. For midwives, a number of factors influence their decision to use continuous CTG monitoring for low risk women. These include a lack of knowledge or skills with IA [8] and being reassured by the sound of the fetal heart in the background [9]. The most often cited reason is fear of medico-legal consequences offset by a perception of continuous CTG as being a protective measure because of the hard copy evidence of monitoring [9] despite midwives’ lack of confidence in its reliability to detect fetal compromise [7,10].

Professional midwifery and obstetric organisations from Australasia, UK, USA and Canada recommend IA of the fetal heart over continuous CTG for low-risk women during labour [11-19]. However, within these guidelines the method recommended in terms of IA frequency, timing and duration of auscultation differs. For example, in the first stage of labour, some recommend listening to the fetal heart every 15 minutes [13,17,18] and others every 15 to 30 minutes [11,12,16,19], whilst in the second stage of labour, the majority recommend listening to the fetal heart every five minutes. In terms of the timing of IA, the majority of guidelines agree, listening to the fetal heart should be conducted after the end of a contraction [11,13,17,19], whilst two guidelines recommend the timing should be during or towards the end of a contraction [12,16]. The recommendations for duration of IA are split between at least 30 seconds [16], 30-60 seconds [11,19], or 60 seconds [13,17,18] in the active stage of labour.

A key driver for this research was an understanding that the evidence supporting the practice of IA of the fetal heart during labour for low-risk women had not been effectively translated into practice. This disconnect between evidence-informed guidelines (what is known) and the decisions informing practice (what gets done) is referred to as the “know-do” gap [20]. This led us to explore why maternity care health professionals are hesitant to use IA as a primary method of fetal heart monitoring for low risk women during labour, despite the existing evidence [1,21] and how we might influence practice change. For this study we decided to develop and test a knowledge translation initiative based on a critical synthesis of the research informed guidelines and underpinned by physiology. We refer to this decision-making framework for IA as Intelligent Structured Intermittent Auscultation (ISIA) [22]. The present paper reports on the implementation of the ISIA framework in one maternity service in New Zealand.

## Methods

This study was a pre-and post-intervention study informed by the science of Knowledge Translation [23] and using the Knowledge-to-Action process [24] to guide the research design. The intervention involved presenting the ISIA decision-making framework during a one-hour education session targeted at maternity care providers in a secondary level maternity service in a major New Zealand city. In this hospital the most common means of fetal heart rate monitoring, both on admission and during labour, was to use the CTG machine.

The study consisted of three phases: the pre-intervention (exploratory) phase, the intervention phase, and post-intervention (explanatory) phase. Retrospective medical record review (RMRR) was the method of data collection in the pre- and post- intervention phases. The RMRR was conducted within an ethical framework: maintenance of patient and staff confidentiality, anonymised information in the final report, no unnecessary data collection, and destruction of data collection forms once they had served their purpose. A confidentiality agreement was signed by all involved in the RMRRs. District Health Board (DHB) and local Māori (indigenous people of New Zealand) approval to conduct the research was granted. Ethical approval for the study was granted by the Health and Disability Ethics Committees (Central Region) New Zealand in November 2009 (CEN/09/10/077).

### Context

In New Zealand all women have a named lead maternity carer (LMC) who coordinates all of their maternity care. Most women (85%) have a midwife LMC providing continuity of care throughout pregnancy, labour and birth and up to six weeks postpartum. The New Zealand midwifery model is a partnership model anchored by informed choice and shared decision-making. LMC midwives (case-loading midwives) may be employed by a hospital or Primary Health Organisation (PHO), or a private provider, or may be self-employed and based in the community. There is a seamless transition between primary, secondary, and tertiary maternity services in New Zealand, based on a comprehensive guideline for referral to obstetric and associated medical specialist care [25]. In some maternity units, employed midwives provide primary maternity care to women unable to access the services of a community LMC midwife, and intrapartum care for women who have a private obstetrician LMC. In this study both hospital employed and self-employed midwives provided midwifery care for women.

#### Phase 1: Pre-intervention

##### Retrospective medical records review (RMRR)

**Figure 1:**
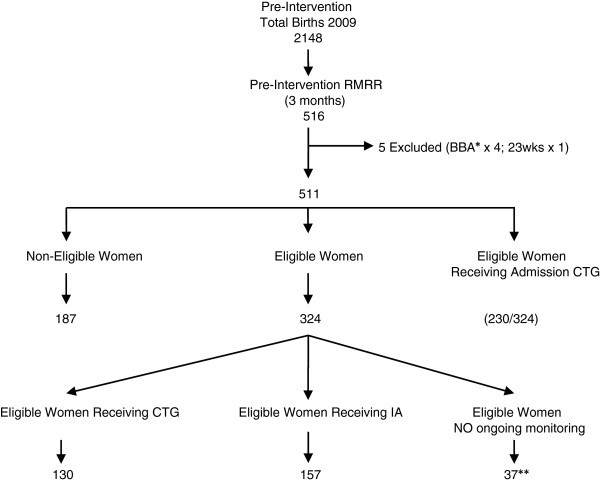
Pre-intervention medical record review: sample distribution.

We obtained baseline data about monitoring practices in an opportunistic sample of women giving birth at the study site between 1 January and 31 March 2009. There were 2148 births in the calendar year 2009 with 188 births in January, 189 in February, and 173 in March giving a potential sample size of 550 births (25% of total births for the year 2009). Of the 550 births in the study time period, we had access to 516 medical records (93.8%), since 34 medical records were in use elsewhere in the hospital and therefore unavailable for the review. Five records were excluded (four babies were born before arrival at hospital and another was 23 weeks gestation) leaving a sample of 511 medical records. The pre-intervention RMRR sample distribution is found in Figure 1.

To establish which women in the sample were suitable to receive IA during labour (i.e. low risk women), the medical records were reviewed for documentation of any antenatal and/or intrapartum risk factors for electronic fetal monitoring, measured against the criteria in the maternity unit fetal surveillance policy. Those records concerning women with no documented risk factors were then scrutinised for evidence of whether or not they received intermittent auscultation. Data collected included: maternal demographics, type of caregiver (LMC/midwife) during labour and birth, admission assessment, use of admission CTG, on-going fetal heart monitoring during labour, mode of birth and neonatal outcomes (Apgar score and admission to Special Care Baby Unit).

Categorisation of monitoring type for data collection purposes was based on the study reported by Cheyne and colleagues in 2003 [26] where continuous CTG was defined as use of the CTG machine for fetal heart monitoring for 75% or more of the labour. For this study, the category, electronic fetal monitoring (EFM), comprised all fetal heart monitoring performed with the CTG machine i.e. continuous CTG, intermittent CTG (short strips of continuous fetal heart monitoring for about 30 minutes throughout active labour) and where a mixture of continuous or intermittent CTG was used throughout the labour care. Admission CTG was defined as approximately 20 – 30 minutes of CTG monitoring conducted at the time of admission. Intermittent auscultation was defined as listening to and counting the fetal heart rate using a Pinard stethoscope or a hand-held Doppler device, following a protocol for frequency, timing and duration.

An online sample size calculator (http://www.raosoft.com/samplesize.html) was used to determine the number of medical records for the medical record review, accepting a 5% margin of error and 95% confidence level. For a population size of 2148, the recommended sample size was 326. Simple descriptive statistics were used to describe the proportion (%) of women suitable for IA during labour, the proportion (%) of women who received IA during labour, compliance with the maternity unit’s IA monitoring guidelines, and the maternal and fetal outcomes when IA was used. For categorical data the chi-squared test was used to determine the significance of any differences in outcomes found between women at low-risk for IA who did or did not receive it. Fisher’s exact test was used if cell values were less than five. We report results as relative risk (RR).

#### Phase 2: Intervention

The intervention involved introducing the ISIA decision-making framework within the maternity unit during a one hour staff education session. Content of the education session included an historical overview of fetal heart monitoring using IA, underpinning physiology influencing fetal heart rate, a review of research and guidelines, and guided critical thinking based on the outcome of robust assessment and examination of the woman at two main decision points during labour.

**Figure 2:**
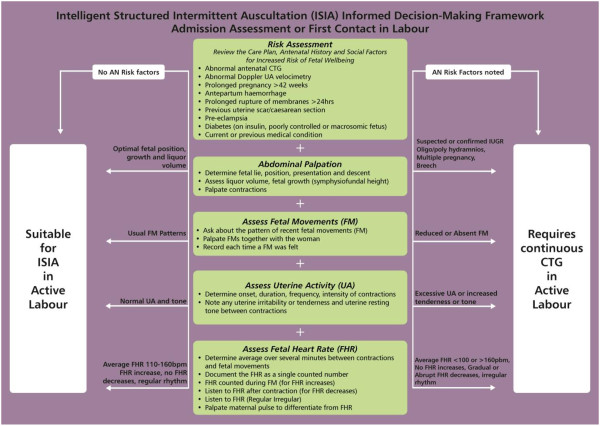
Intelligent Structured Intermittent Auscultation (ISIA) Informed Decision-Making Framework for Admission Assessment or First Contact in Labour.

**Figure 3:**
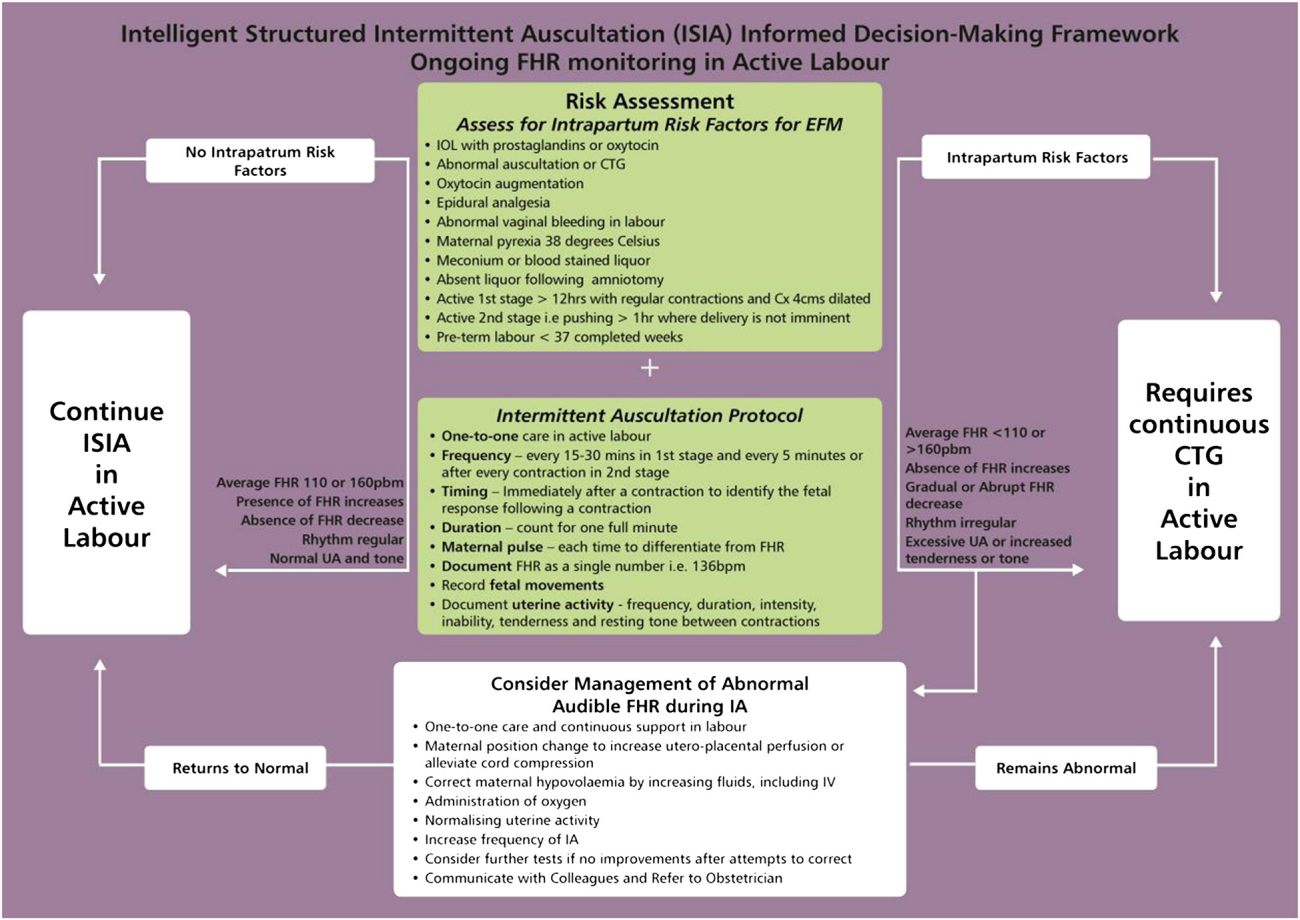
Intelligent Structured Intermittent Auscultation (ISIA) Informed Decision-Making Framework for Ongoing Fetal Heart Rate Monitoring in Active Labour.

The ISIA framework was influenced by a critical synthesis of research evidence, clinical guidelines, and with feedback during 2007 and 2010 from midwifery experts on three international, on-line discussion groups of which we are members (nzmidwives@yahoogroups.com; Midwifery-Reasearch@jiscmail.ac.uk and Normalbirth-Research@Jiscmail.ac.uk). Disussion was triggered by asking midwives their opinions around the frequency, timing and duration of IA. There were 55 postings from 31 midwives from New Zealand, Australia, UK, USA, and Europe with discussions around barriers to the use of IA in practice. These included the role of evidence/guidelines, staffing levels impacting on midwives’ ability to perform IA, EFM used as a defensive practice, and questioning the accuracy/variability of IA. There was widespread agreement for using normal physiology as the starting point for understanding and interpreting IA along with the use of fetal movements in conjunction with IA as an indicator of fetal well-being. The midwives highlighted a need for a tool or strategy that contained information to assist clinicians develop an understanding of the physiology behind FHR monitoring and to facilitate the successful teaching and implementation of IA in practice.ISIA was developed by the authors as an algorithm for use at the admission assessment, or first contact in labour, to determine what monitoring approach is suitable for the individual low risk woman for ongoing fetal heart monitoring in active labour. The detailed algorithms are presented in Figures 2 and 3.

The intervention was structured to include both direct and indirect contact with those health professionals who would be called upon to monitor the fetal heart rate. Direct contact consisted of a formal face-to-face instructional presentation of the ISIA framework, and indirect contact consisted of posters and informational material about ISIA made available in the unit. Direct participants were recruited through invitations and posters in the maternity unit. There were potentially just over 100 midwifery and medical staff working in or with access to the maternity facility at the time of the delivery of the intervention with 33 accepting the invitation to participate.

ISIA was thus introduced directly to 33 individuals (made up of 15 hospital employed midwives [30% of all hospital employed midwives], 14 self-employed midwives [36% of all self-employed midwives] and four doctors (33% of all doctors in the unit) including two obstetric registrars and two senior house officers). ISIA was introduced in four, one-hour education sessions, over a two week period, at staff handover time (2.30 pm – 3.30 pm). The session included a short presentation with slides and printed hand-outs. Additional information provided to participants included the New Zealand College of Midwives (NZCOM) fetal monitoring consensus statement [14]. All sessions were interactive, with questions from the participants mostly based on real-life scenarios from practice, and requests for clarification. This interaction offered an opportunity to further highlight the evidence around the use of IA for low-risk women.

Indirect introduction of the ISIA framework also occurred through opportunistic viewing of a DVD of the education session made available for staff to look at during quiet times at work or independently. In addition, posters describing the ISIA framework were placed in the workstations in the delivery suite and the wards. These posters provided visual prompts for midwives to initiate discussion with those members of staff who had not been able to attend the education session. Senior staff used the posters and hand-outs during staff handover time to further highlight the evidence for IA as the recommended fetal monitoring modality for low risk women and to challenge decisions for non-clinically indicated admission CTG and/or continuous CTG.

##### Phase 3: Post-intervention

**Figure 4:**
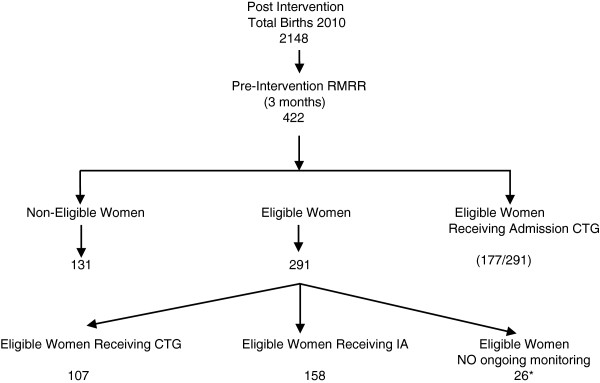
Post-intervention medical record review: sample distribution.

The post-intervention phase aimed to determine whether there were changes in fetal heart rate monitoring practices for low-risk women following the intervention. A further RMRR was conducted between three and six months after the education session which introduced the ISIA framework. The post-intervention RMRR took place over a three month period from 1 April to 30 June, 2010. There were again 2148 births in the calendar year 2010, with 177 in March, 167 in April, and 170 in May, giving a potential sample size of 514 births. This represents 24% of total births for the year 2010. Of the 514 births in the study time period, we had access to 422 medical records (82.1%). The lower rate of medical record availability in the post-intervention phase was related to an inability to access 92 medical records involved in another clinical audit occurring simultaneously during the study period. Post-intervention RMRR sample distribution is found in Figure 4. The same process for assessing suitability for IA used in the pre-intervention RMRR was again used. The sample size calculator previously described was again used to calculate a required sample of 326.

## Results

The findings are presented as comparisons of demographic distribution, admission assessment criteria, admission CTG and maternal and neonatal outcomes, between women who received IA and women who received CTG monitoring during labour, with the exception of findings related to the conduct of IA. Findings related to how IA was conducted are compared by pre- and post-intervention.In the pre-intervention phase, a total of 511 records were reviewed, of which 324 (63.4%) were for low-risk women. Main exclusion criteria were previous caesarean section, hypertension, preeclampsia and other medical conditions, suspected intra uterine growth restriction (IUGR) and post-term pregnancy. Of these 324 low-risk women, 157 (48.5%) received IA for ongoing fetal heart monitoring during active labour, whilst 130 (40.1%) received continuous CTG monitoring. Thirty seven (11.4%) low risk women had no ongoing fetal heart monitoring during active labour, mainly due to giving birth soon after admission (Figure 1).A total of 422 medical records were reviewed in the post-intervention phase, of which 291 (69%) were for low risk women. In this phase 158 (54.3%) low risk women received IA for ongoing fetal heart monitoring during active labour and 107 (36.8%) received continuous CTG monitoring. Twenty six (8.9%) low risk women had no ongoing monitoring due to giving birth soon after admission (Figure 4).

The women who received intermittent auscultation and the women who received continuous CTG did not differ on any demographic characteristic in the pre- or post-intervention medical record review samples as described in Table 1.

**Table 1:** A comparison of the demographic distribution of the pre and post intervention samples

		**Pre –intervention medical record review**	**Post–intervention medical record review**	**Pre–intervention medical record review**	**Post–intervention medical record review**
		**Eligible women receiving IA**	**Eligible women receiving IA**	**Eligible women receiving CTG (Continuous or Intermittent)**	**Eligible women receiving CTG (Continuous or Intermittent)**
		**n = 157**	**n = 158**	**n = 130**	**n = 107**
		**Number (%)**	**Number (%)**	**Number (%)**	**Number (%)**
**Ethnicity**	NZ European	66 (42)	73 (46.2)	62(47.7)	51 (47.7)
	Maori	30 (19.1)	28 (17.7)	18 (13.8)	14 (13.1)
	Pacific People	12 (7.6)	12 (7.6)	7 (5.4)	6 (5.6)
	Asian	8 (5.1)	9 (5.7)	9 (6.9)	10 (9.4)
	Indian	3 (1.9)	3 (5.7)	1 (0.8)	4 (3.7)
	African	1 (0.6)	1 (0.6)	2 (1.5)	1 (0.9)
	Other	4 (2.5)	2 (1.3)	2 (1.5)	4 (3.7)
	Not stated	33 (21)	30 (19)	29 (22.3)	17 (15.9)
**Gravidity**	Primigravid	53 (33.7)	48 (30.4)	59 (45.5)	45 (42)
	Multigravid	104 (66.3)	110 (69.6)	71 (56.5)	62 (58)
**Parity**	Nulliparous	62 (39.5)	67 (42.4)	77 (59.2	66 (61.7)
	Multiparous	95 (60.5)	92 (58.2)	53 (40.8)	41 (38.3)
**Gestation**	Range	36 wks + 1 day to 41 wks + 5 days	37 wks to 42 wks	36 wks + 3 days to 42 wks + 2 days	36 wks + 3 days to 42 wks + 2 days
	Mean	278.9 days (39 wks +1 day)	279.9(39 wks + 5 days)	279.5 (39 wks + 6 days)	279.5 (39 wks + 6 days)
	SD	± 7.5 days	±7.039 days	± 9.172	± 9.172
**Midwife during labour**	Self-Employed Midwife	131 (83.4)	146 (92.4)	82 (63.1)	84 (78.5)
	*Hospital Midwife	26(16.6)	12 (7.6)	48 (37.9)	23 (21.5)

*Hospital midwives provide midwifery care during labour for hospital primary women and private obstetricians.

### Admission CTG and admission assessment criteria

In this group of women, the risk of receiving an admission CTG was significantly reduced following the intervention (RR 0.75, 95% CI, 0.60 – 0.95, p = 0.016). There was a risk benefit for all women of increased recordings of maternal pulse in conjunction with fetal heart monitoring during admission assessment (IA group - RR 4.54, 95% CI, 2.06 – 9.98, p = 0.0002; CTG group - RR 4.10, 95% CI, 1.94 – 8.64, p = 0.0002). There was an increase in documenting fetal heart rate during a fetal movement (Fisher’s Exact p = .015) in the post-intervention phase. However, whilst there was a small increase in the recording of fetal movements on admission, this result failed to reach statistical significance (Table 2).

**Table 2:** Comparison of admission assessment criteria for eligible women by monitoring type pre- and post- intervention

	**Pre –intervention medical record review**	**Post–intervention medical record review**	**Significance**	**Pre –intervention medical record review**	**Post–intervention medical record review**	**Sig.level**
**Admission assessment criteria**	**Eligible women receiving IA**	**Eligible women receiving IA**		**Eligible women receiving CTG**	**Eligible women receiving CTG**	
	**n = 157/%**	**n = 158/%**		**n = 130/%**	**n = 107/%**	
**Admission CTG**						
Yes	88 (56.1)	67 (42.4)	RR 0.75, 95% CI, 0.60 – 0.95, p = 0.016	120 (92.3)	99 (92.5)	NS
**Abdominal palpation**						
Yes	89 (56.7)	82 (51.9)	NS	80 (61.5)	63 (58.9)	NS
**Fetal lie**						
Yes	67 (42.7)	56 (35.4)	NS	59 (45.4)	46 (43)	NS
**Fetal position**						
Yes	72 (45.8)	67 (42.4)	NS	65 (50)	57 (53.3)	NS
**Fetal presentation**						
Yes	83 (52.9)	70 (44.3)	NS	75 (57.7)	57 (53.3)	NS
**Fetal descent**						
Yes	67 (42.7)	54 (34.2)	NS	61 (47)	47 (43.9)	NS
**Fetal movement (FM) patterns**						
Yes	25 (15.9)	32 (20.3)	NS	25 (19.2)	21 (20)	NS
**FM palpated by midwife and woman**						
Yes	0 (0)	3 (1.9)	NS	4 (3.1)	3 (2.8)	NS
**FHR heard during FM**						
Yes	0 (0)	7 (4.4)	Fisher’s Exact p = .015	1 (0.8)	3 (2.8)	NS
**Uterine activity**						
Yes	99 (63.1)	115 (72.8)	NS	71 (54.6)	69 (64.5)	NS
**Contraction frequency**						
Yes	109 (69.4)	97 (61.4)	NS	61 (47)	49 (45.8)	NS
**Contraction Duration**						
Yes	38 (24.2)	36 (22.8)	NS	18 (13.8)	12 (11.2)	NS
**Contraction strength**						
Yes	59 (37.6)	55 (34.8)	NS	31 (23.8)	30 (28)	NS
**Uterine resting tone**						
Yes	1 (0.6)	0 (0)	NS	0 (0)	2 (1.9)	NS
**FHR documented as a single number**						
Yes	67 (42.7)	83 (52.5)	NS	50 (38.5)	44 (41.1)	NS
**Maternal pulse noted**						
Yes	7 (44.5)	32 (20.3)	RR 4.54 95% CI, 2.06 – 9.98, p = 0.0002	8 (6.2)	27 (25.2)	RR 4.10 95% CI, 1.94 – 8.64, p = 0.0002
**FH rhythm noted**						
Yes	1 (0.6)	3 (1.9)	NS	0 (0)	6 (5.6)	Fisher’s Exact p = .008
**Accelerations noted**						
Yes	61 (38.8)	33 (20.9)	RR 1.29 95% CI 1.11 – 1.50, p = <0.0007	59 (45.4)	38 (35.5)	NS
**Decelerations noted**						
Yes	39 (24.8)	26 (16.5)	NS	47 (36.2)	35 (32.7)	NS

### On-going intermittent auscultation and documentation

Following the intervention, there was an increase in the use of IA during labour represented by a relative change of 12% (result not shown), with improved documentation of clinical findings from assessments, and a significant reduction in the risk of receiving an admission CTG (RR 0.75, 95% CI, 0.60 – 0.95, p = 0.016).

For women receiving IA during active labour, medical records demonstrated a reduced risk of not having the FH auscultated after a contraction (RR 0.81, 95% CI, 0.69 - 0.95, p = 0.009). Also in this group of women, there was a reduced risk of not having the auscultated fetal heart rate recorded as a single number (as opposed to a range of numbers, such as, “FHR 130-146”) (RR 0.60, 95% CI, 0.50 - 0.72, p = < 0.0001). There was reduced risk of not recording uterine contraction frequency (RR 0.84, 95% CI, 0.72 - 0.98, p = 0.034), strength (RR 0.83, 95% CI, 0.73 - 0.95, p = 0.007) and duration (RR 0.92, 95% CI, 0.86 - 0.98, p = 0.012). Following the intervention there was a reduced risk of not auscultating the fetal heart every 15 to 30 minutes, as stated in the hospital protocol (RR 0.81, 95% CI, 0.66 -1.00, p = 0.05) There were no changes in the way in which fetal movements during labour were recorded or described (Table 3).

**Table 3:** Comparison of compliance with hospital IA protocol and documentation requirements during ongoing monitoring with IA pre- and post- intervention

	**Pre –intervention RMRR**	**Post–intervention RMRR**	**Significance**
**IA Protocol**	**Eligible women receiving IA**	**Eligible women receiving IA**	
	**n = 157**	**n = 158**	
**Frequency 1**^**st **^**stage 15-30 mins**			
Yes	76 (48.4)	94 (59.5)	RR 0.81, 95% CI, 0.66 -1.00, p = 0.05
**Frequency 2**^**nd **^**stage every 5 mins**			
Yes	69 (44)	77 (48.7)	NS
**Timing (after contraction)**			
Yes	40 (25.5)	62 (39.2)	RR 0.81, 95% CI, 0.69 - 0.95, p = 0.009
**Duration (for 1 minute)**			
Yes	16 (10.2)	23 (14.6)	NS
**Maternal pulse**			
Yes	1 (0.6)	5 (3.2)	NS
**FH Written as a single number**			
Yes	33 (21)	82 (51.9)	RR 0.60, 95% CI, 0.50 - 0.72, p = < 0.0001
**FH rhythm**			
Yes	0 (0)	3 (1.9)	NS
**Accelerations**			
Yes	9 (5.7)	17 (10.7)	NS
**Decelerations**			
Yes	11 (7)	16 (10.1)	NS
**Fetal movements any time during labour**			
Yes	7 (4.5)	6 (3.8)	NS
**Uterine activity frequency**			
Yes	42 (26.8)	60 (38)	RR 0.84, 95% CI, 0.72 - 0.98, p = 0.034
**Uterine activity strength**			
Yes	28 (17.8)	49 (31)	RR 0.83, 95% CI, 0.73 - 0.95, p = 0.007
**Uterine activity duration**			
Yes	6 (3.8)	18 (11.4)	RR 0.92, 95% CI, 0.86 - 0.98, p = 0.012
**Uterine resting tone**			
Yes	2 (1.3)	3 (1.9)	NS

### Birth outcomes

Vaginal birth was higher for all low risk women who had IA monitoring during labour (pre and post intervention combined 94%) compared with low risk women who received continuous CTG monitoring during labour (pre and post intervention combined 79%). There was a reduced risk of caesarean section across the combined pre- and post-intervention samples for women monitored with IA (RR 0.29, 95% CI, 0.17 – 0.48, p = <0.0001).

Twenty four babies of women who received continuous CTG compared with 18 babies of women who received IA required admission to the Special Care Baby Unit (Table 4), representing an absolute difference of 4.4%. This was not a statistically significant finding (RR 0.56, 95% CI 0.31 - 1.01, p = 0.056). Only one neonate in each of the pre- and post-intervention samples had a five minute Apgar score below 7.

**Table 4:** Comparison of maternal and neonatal outcomes pre- and post- intervention

	**Pre –intervention medical record review**	**Post–intervention medical record review**		**Pre –intervention medical record review**	**Post–intervention medical record review**	
	**Eligible women receiving IA**	**Eligible women receiving IA**	**Significance**	**Eligible women receiving CTG (Cont. or Int.)**	**Eligible women receiving CTG (Cont. or Int.)**	**Significance**
	**n = 157**	**n = 158**		**n = 130**	**n = 107**	
	**N (%)**	**N (%)**		**N (%)**	**N (%)**	
**Mode of birth**						
Vaginal (normal and assisted)	145 (92.4)	151 (95.6)	NS	108 (83.1)	80 (74.8)	NS
CS (acute and elective)	12 (7.6)	7 (4.4)	NS	22 (16.9)	27 (25.2)	
**Admission to SCBU/NICU**						
Yes	10 (6.4)	8 (5.1)	NS	8 (5.1)	16 (10.1)	RR 0.41, 95% CI, 0.18 - 0.92, p = 0.03
**Apgar score < 7@5 mins**						
Yes	1	1	NS	1	1	NS

## Discussion

This study addressed the clinical problem of exposing low risk birthing women to the unnecessary use of CTG monitoring, both on admission to hospital and continuously during labour. The choice and use of IA for fetal heart monitoring has been largely displaced by the ubiquitous availability of technology in the modern birthing room, together with the increased use of epidural anaesthesia and synthetic oxytocin in ‘normal’ birthing women [27]. Arguably, the displacement of IA has implications midwifery practice and the safety of mothers and babies since CTG monitoring increases the likelihood of operative birth with increases in maternal and neonatal morbidity. As a consequence we proposed that IA should be re-established as a fundamental midwifery skill and offered to low risk women as a safe and effective alternative to CTG monitoring. Few robust IA guidelines with established validity and reliability exist to guide the practice of IA [1,28]. Therefore the ISIA framework, developed for this purpose, was evaluated in this pre-post intervention study.

The ISIA decision-making framework for fetal heart monitoring, overtly informed by an understanding of fetal physiology and research evidence, is a knowledge translation innovation developed to guide maternity care providers in their decision making regarding fetal heart monitoring choice, clinical practice, and interpretation. The new ISIA framework has two parts: “Admission Assessment or First Contact in Labour” and “Ongoing IA in Active Labour”. The findings are discussed in the context of these two parts of the ISIA framework.

### ISIA for admission assessment or first contact in labour

Labour is one part of the whole childbearing continuum from conception to discharge at six weeks [6] and risk factors may develop at any stage throughout pregnancy. A thorough examination and assessment of the woman on admission to hospital or at the first contact in labour (which could be at home) will help midwives to determine whether there are risk factors, either previously present or recently developed, that signal potential for fetal compromise during labour. This determination of the woman’s risk status represents a key decision point in the choice of fetal heart monitoring modality [29].

In the hospital setting, it is at this admission assessment that some maternity care providers believe an admission CTG is justified; and indeed many midwives and doctors still recommend and use this technology despite a lack of evidence supporting its use for low-risk women [4,5]. For some women, the admission CTG becomes continuous because staff are too busy to take off the monitor or there are not enough midwives to provide one-to-one care and the CTG becomes a ‘baby-sitter’ [29].

The baseline findings from this study confirmed that low risk women were unnecessarily exposed to CTG monitoring as reported by other authors [29-33] with over half of all women receiving an admission CTG. However, following the intervention, the risk of receiving an admission CTG was significantly reduced. The ISIA framework provided guidance to midwives’ decision-making.

### ISIA in active labour

The second key decision point associated with choice of fetal heart monitoring modality occurs after the initial assessment has been completed and the woman’s risk status determined [29]. The collective findings from the assessment are discussed with the woman and a decision about FHR monitoring modality can be made and documented on the care plan. In the absence of any risk factors and when all other parameters of the ISIA admission assessment are normal, it is appropriate to offer and recommend intermittent auscultation for ongoing FHR monitoring during labour, and a statement to this effect is entered in the woman’s medical record [22].

A change in midwives’ practice was evidenced by a relative change 12% in the use of IA for ongoing fetal heart monitoring during labour following the intervention. This increased use of IA complies with evidence-based guidelines for fetal monitoring for low risk women [11-19]. Other important changes following the intervention were demonstrated in the recording of maternal clinical findings associated with high quality fetal heart monitoring. These included the recording of maternal pulse rate, contraction frequency, strength and duration and writing the fetal heart rate as a single number as opposed to a range of numbers.

With IA and continuous CTG monitoring, it is vital to differentiate between maternal heart rate and fetal heart rate [1,6,34]. Serious adverse outcomes have been reported when this differentiation has not been identified. This is especially relevant in the case of increased fetal activity, poor placement of the CTG transducer, and maternal tachycardia due to infection or medications such as those used to prevent pre-term labour, or during second stage of labour. There are increasing reports of CTG machines recording what appears to be a ‘normal’ fetal heart rate with the subsequent birth of a severely compromised or stillborn baby [6,34]. In this study the ISIA framework and the teaching associated with its use during labour appears to have influenced midwives’ understanding and practice around this very important aspect of care and fetal monitoring.

Simultaneous auscultation of the fetal heart and palpation of uterine contractions are necessary to evaluate fetal well-being [1,6]. Whilst there was improvement in the documentation of contraction frequency, timing and duration following the intervention, there is still room for improvement in documentation of uterine tenderness, irritability and resting tone between contractions. Midwives were more likely to auscultate the fetal heart every 15 to 30 minutes, as required by the hospital protocol following the intervention however, there was no change in the way in which fetal movements were recorded or described during labour. There is a dearth of research investigating a link between fetal movement and fetal well-being during labour, which may have contributed to it not being incorporated into practice at this stage. Studies of antenatal fetal movement patterns [35-37] might potentially be extrapolated to labour care, but this needs further investigation.

One of the criticisms of IA as a monitoring modality is that fetal heart rate variability is unable to be ascertained simply by listening and counting. Determination of baseline variability is associated with continuous CTG monitoring and fluctuations of 15 beats per minute or more from the baseline rate are considered a marker of fetal well-being. CTG monitoring is the recommended monitoring modality for women with complicated pregnancies associated with a higher risk of fetal compromise. For well women with uncomplicated pregnancies and well grown fetuses, the likelihood of fetal compromise is less, therefore determination of fetal heart rate variability is not required. The relevance of baseline variability to this study is in the way in which midwives document the fetal heart rate (FHR) when using IA. As IA is a listening and counting method (counted over one minute) the FHR should be recorded as a single number, such as when the maternal pulse is documented. Many midwives, especially those using a hand-held Doppler device with a digital display of numbers, document the FHR as a range of numbers such as FHR 130-146, in the mistaken belief this demonstrates baseline variability (and by default, a higher quality of FHR monitoring).

The ISIA framework encourages midwives to make decisions about fetal well-being by assessing for fetal heart rate increases associated with fetal movements and following contractions. An increase in the FHR above the pre-determined average FHR at these times provides reassurance of fetal well-being. Midwives are encouraged to use the findings from their assessment using ISIA rather than converting what they understand from CTG monitoring to the interpretation of IA findings. ISIA encourages the midwife to document the FHR as a single number. Following the intervention the medical records demonstrated a reduced risk of not recording the auscultated FHR as a single number.

### Limitations

This research was conducted in only one New Zealand maternity unit, and as such may not be generalisable to other maternity units. However, the idea of fittingness [38] may be more appropriate to consider. Fittingness is described as the findings ‘fitting’ the context outside the current study site or when the reader/practitioner considers the findings as applicable and meaningful in terms of their own experience [39].

The use of the clinical record as the main source of data for this study has potential limitations such as availability, accessibility, adequacy, veracity and completeness. However, medical record review is a widely used method of data collection in health disciplines for the assessment of knowledge use and quality improvement in particular. Most records were available and accessible. Data extraction was undertaken by a specially trained midwife audit team to ensure veracity and completeness of the data. Length of follow up may be an issue since the post intervention audit was conducted three - six months after the delivery of the intervention. A longer follow up would have revealed long- term sustainability of practice change; although the final data was collected at six months after the intervention. A time series analysis would reveal any decay in the practice over time. This would need to be addressed in any future study of this intervention.

## Conclusion

The ISIA decision-making framework incorporates clinical skills and indicators that include listening to the fetal heart, into one framework. The framework establishes the importance of all elements of care that make up ISIA, which is its point of difference from usual practice. Even though IA has been around a long time, ISIA is asking something new of practitioners in relation to auscultating the fetal heart.

The ISIA informed decision-making framework supported midwives to make changes to their practice, with a higher adherence to the use of intermittent auscultation as one component of the admission assessment, and for ongoing fetal heart monitoring in active labour. ISIA has provided this group of midwives with a means of systematically assessing each woman and fetus in labour and to document their findings during the assessment in a manner that demonstrates their critical thinking and clinical reasoning.

Most fetal surveillance guidelines simply provide a protocol for intermittent auscultation outlining the frequency, timing and duration of intermittent auscultation. We recommend that fetal monitoring guidelines be amended to include a more comprehensive description of IA using the ISIA framework for admission assessment and ongoing FHR monitoring during labour.

## Competing interests

The authors declare there are no competing interests.

## Authors’ contributions

RMM, MJF and JPS conceived the idea for researching IA in the context of keeping birth normal. RMM, MJF and JPS were involved in the study design. RMM developed the intervention (ISIA framework and education session) implementation, and analysis. RMM prepared the first and subsequent drafts, and all authors reviewed and approved various drafts and the final paper. All authors read and approved the final manuscript.

## Authors’ information

RMM: Senior lecturer, PhD in Midwifery, MA (Applied) Midwifery, BN, RM, RN.

JPS: Senior lecturer, PhD in Midwifery, MA (Applied), RM, RN.

MJF: Professor of Midwifery, PhD in Midwifery, Graduate Diploma in Clinical Epidemiology and Biostatistics; BA (Psychology and Sociology), RM, RN.

## Pre-publication history

The pre-publication history for this paper can be accessed here:

http://www.biomedcentral.com/1471-2393/14/184/prepub
